# Insect-Mimetic Imaging System Based on a Microlens Array Fabricated by a Patterned-Layer Integrating Soft Lithography Process

**DOI:** 10.3390/s18072011

**Published:** 2018-06-22

**Authors:** Minwon Seo, Jong-Mo Seo, Dong-il “Dan” Cho, Kyo-in Koo

**Affiliations:** 1 Department of Electrical and Computer Engineering, Seoul National University, Seoul 08826, Korea; sm7bmw@snu.ac.kr (M.S.); dicho@snu.ac.kr (D.C.); 2 Biomedical Research Institute, Seoul National University Hospital, Seoul 03080, Korea; 3 Department of Biomedical Engineering, University of Ulsan, Ulsan 44610, Korea

**Keywords:** microlens array, insect-mimetic, multiaperture vision system

## Abstract

In nature, arthropods have evolved to utilize a multiaperture vision system with a micro-optical structure which has advantages, such as compact size and wide-angle view, compared to that of a single-aperture vision system. In this paper, we present a multiaperture imaging system using a microlens array fabricated by a patterned-layer integrating soft lithography (PLISL) process which is based on a molding technique that can transfer three-dimensional structures and a gold screening layer simultaneously. The imaging system consists of a microlens array, a lens-adjusting jig, and a conventional (charge-coupled device) CCD image sensor. The microlens array has a light screening layer patterned among all the microlenses by the PLISL process to prevent light interference. The three-dimensionally printed jig adjusts the microlens array on the conventional CCD sensor for the focused image. The manufactured imaging system has a thin optic system and a large field-of-view of 100 degrees. The developed imaging system takes multiple images at once. To show its possible applications, multiple depth plane images were reconstructed based on the taken subimages with a single shot.

## 1. Introduction

Most conventional imaging systems adopt a single aperture structure inspired by vertebrates’ vision system. Stacking lenses along the optical axis enables this imaging system to achieve a wide-angle view, a telescopic view, and reduced aberrations. However, the single-aperture vision system has disadvantages, such as the limitation of miniaturization and low time resolution [1]. To overcome these disadvantages, efforts have been made to mimic insects’ vision system.

**Figure 1:**
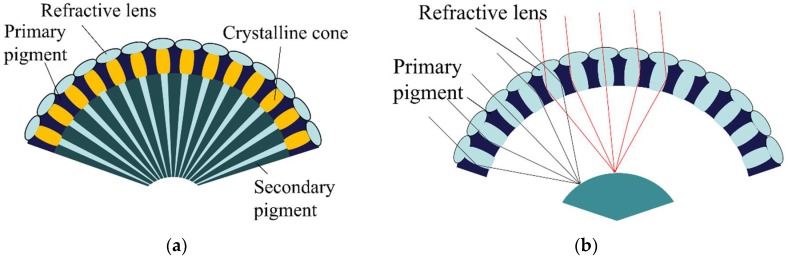
The schematic diagram of (**a**) the apposition compound eye and (**b**) the superposition compound eye.

Figure 1 shows a schematic illustration of anatomical structures of the insect compound eye. There are two types of compound eye: an apposition compound eye and a superposition compound eye [2]. The apposition compound eye receives light through hundreds to thousands of facets. Every facet has a pigment to prevent light from entering neighborhood facets. This enables a distinct point pixel instead of a cross-talked white Gaussian noised pixel [3,4]. Oppositely, insects with a superposition compound eye, which are those insects that usually move around at nighttime, do not have the pigment unit among the facets. This pigmentless structure causes light interference on photoreceptors and blurs object images. However, superposed light in the pigmentless facets enables the nocturnal insect to react more sensitively to the nighttime environment [5,6].

To mimic the structure of the compound eye, a microlens array has been widely used [7,8,9,10]. To fabricate the microlens array, a thermal reflow process is usually employed because of its reliable spherical profile as well as its simple fabrication process.

There are several works mimicking the compound eye of *Xenos peckii*, which envisions subimages of the object with its clustered eyes [11,12,13]. To perceive high-quality subimages from the compound eye, a light screening layer among the microlenses is indispensable. Morgan et al. [14] implemented an opaque wall to change the color of glasses among microlenses using thermal treatment. Ichioka et al. [15] inserted optical separators between an image sensor and a microlens array. This method required aligning process of the optical separator to the microlenses. Kim et al. [16] made a perforated black polymer sheet and covered a curved microlens array with the black sheet to block the interference of light. The abovementioned techniques used to achieve a microlens array with a screening layer require manual alignment or high thermal treatment.

In this paper, we presented a compound eye mimicking an imaging system using a microlens array integrated with a gold screening layer fabricated by a patterned-layer integrating soft lithography (PLISL) process. The microlens array integrated with the light screening pattern was adjusted on a conventional charge-coupled device (CCD) image sensor by a lens-adjusting jig. The captured image was refocused in three different planes to demonstrate depth perception capability. Moreover, in order to demonstrate the extensibility of our PLISL process, a microlens array with a gold screening layer was fabricated on a spherical surface as well.

## 2. Materials and Methods

### 2.1. Fabrication of a Microlens Array with a Gold Screening Layer

In order to determine the focal length of the microlens, the thickness of the glass protecting the image sensor should be considered. The thickness of the protecting glass, as measured by a caliper, was 0.44 mm. It was assumed that the glass has a refractive index similar to N-BK7 glass, which has a refractive index of 1.517. Therefore, the focal length of the microlens should be above 0.67 mm.

Since the thermal reflow process enables a cylindrical photoresist structure to transform into a microlens structure due to the property of minimizing surface energy [17], the height (H) and diameter (2r) of the cylindrical structure decides the curvature of the microlens. The height of a cylinder with 10 μm and a refractive index of UV curing resin is 1.56 [18]. Therefore, the height of the microlens can be calculated by Equations (1) and (2). The radius of the microlens should be above 118 μm. In this paper, the radius of the microlens array was set to 175 μm for the thin optical system and stable focus adjustability (See Supplementary Information).

(1) $$\pi r^{2}H=\;\frac{\pi h}{6}\left(3r^{2}+h^{2}\right)$$

(2) $$h=\;\sqrt[{3}]{X}-\;\frac{r^{2}}{\sqrt[{3}]{X}}\;for\;X=\;\sqrt{9H^{2}r^{4}+r^{6}}+3Hr^{2}$$

(3) $$f=\;\frac{r^{2}+h^{2}}{2h\left(n-1\right)}$$

**Figure 2:**
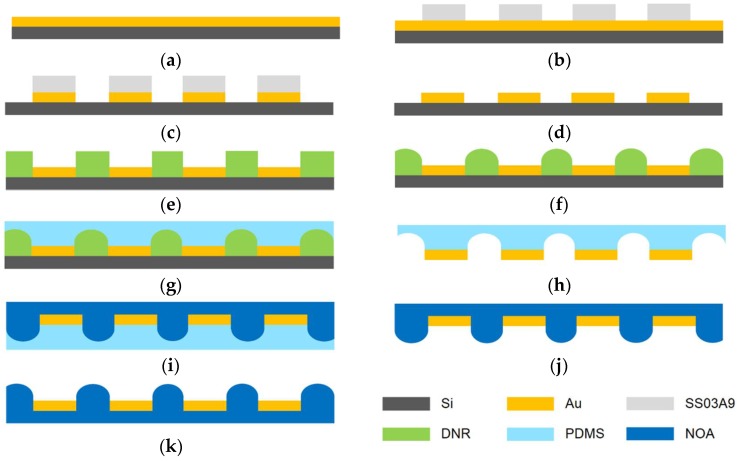
The specific steps of the PLISL process. (**a**) The gold layer was deposited on the silicon; (**b**) positive PR patterning; (**c**) gold layer etching; (**d**) PR removal; (**e**) negative PR patterning; (**f**) thermal reflow; (**g**) 1st molding with the elastomer; (**h**) the 1st molded layer; (**i**) 2nd molding with the UV resin; (**j**) the 2nd molded layer; (**k**) the fabricated microlens array with gold layer.

The soft lithography process is widely used to fabricate three-dimensional microstructures. Even though the soft lithography process has several advantages, including repeatability and simplicity, it is not easy to add another patterned layer on the surface of the soft-lithography-processed polymer. In this investigation, to fabricate a screening layer among the microlenses, a PLISL process was proposed, as shown in Figure 2. Basically, the proposed process was an advanced version of the soft lithography process. A gold film which has weak adhesion to a silicon wafer [19] was used as a light screening material. It was patterned on a silicon substrate without any adhesion-promoting layer like titanium or chrome. On the gold prepatterned substrate, a three-dimensional microstructure was implemented and transferred to the polymer material by molding. During the molding process, the prepatterned gold film was transferred and integrated onto the polymer because of its low adhesion to the silicon wafer.

The specific steps of the PLISL process are as follows: A silicon wafer was cleaned in a mixture of H_2_SO_4_:H_2_O_2_ (3:1) for 30 min at 60 degrees Celsius. After that, a 120-nm thick gold layer [20], which would serve as the light interference screening layer, was deposited on the cleaned wafer by an E-gun evaporator (ZZS550-2/D, Maestech Co., Ltd., Pyungtaek, Korea) without any adhesion layer like titanium or chrome (Figure 2a). A positive photoresist, SS-03A9 (Dongwoo Fine-chem Co., Ltd., Gyeonggi-do, Korea), was patterned on the gold evaporated silicon wafer (Figure 2b). The photoresist patterned wafer was etched by gold etchant, which was a mixture of I_2_:KI:H_2_O (1:4:10) (Figure 2c). After removing the photoresist on the wafer with acetone, the gold-patterned wafer was coated by negative photoresist, DNR (Dongjin Semichem Co., Ltd., Incheon, Korea), about 10 μm by a spin coater and baked on a hot plate at 95 degrees Celsius. The second photoresist layer was coated on the first photoresist layer and baked on the hot plate at 110 degrees Celsius. The wafer was exposed using the identical mask with the gold-layer patterning mask to pattern on the gold-free area (Figure 2e). After the UV exposed wafer was developed, the developed wafer was baked on a hot plate to melt the photoresist pattern for the spherical structure by the surface tension (Figure 2f). This heating step is widely known as the thermal reflow process.

After the silicon substrate steps, the microlens array pattern and the gold screening layer was transferred using polydimethylsiloxane (PDMS, Sylgard 184, Dow Corning, Scottsdale, AZ, USA) (Figure 2h). To get a convex microlens array with a screening layer, a UV-curable epoxy resin (NOA 72, Norland Products Inc., Cranbury, NJ, USA) with a refractive index 1.56 was poured on the concave-patterned PDMS mold. A transparent polyethylene-terephthalate (PET) film was placed on the poured epoxy resin for flattening and easy handling. The resin was cured by 15 W UV light with a wavelength of 365 nm (Figure 2i). The cured and stripped resin had a convex microlens array integrated with the gold layer, as shown in Figure 2k. Finally, a thin parylene was deposited on the convex microlens array and the gold screening layer for electrical isolation from the CCD sensor.

### 2.2. Imaging System Setup Using a 3D Printed Jig

**Figure 3:**
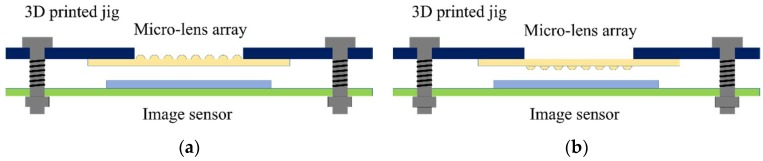
The schematic diagram of the imaging system setup. (**a**) The convex part of the microlens array looks outside. (**b**) The convex part of the microlens array faces the CCD sensor.

Figure 3 shows a schematic of the proposed imaging system. It consists of a microlens array, a focus-adjustable jig, and a 6.72-mm CCD sensor (1920 × 1080 pixels, P5756, Hanjindata, Gyeonggi-do, Korea). The jig was designed and three-dimensionally printed for secure positioning and focusing of the microlens array. The aperture size of the jig was 4 mm. Screws and springs were used to adjust a four-point focal distance between the microlens array and the CCD sensor.

There are two possible directions when integrating the microlens array with the sensor. One is that a convex part of the microlens array looks outside (Figure 3a). The other is that the convex part of the microlens array faces the CCD sensor (Figure 3b). In the case of the microlens array facing the CCD sensor, the gold screening layer is closer to the CCD sensor compared with the case of the microlens array looking outside. This closer distance is expected to reduce the interference of the entered light and to enhance imaging quality.

## 3. Results

### 3.1. Characteristics of the Microlens Array

**Figure 4:**
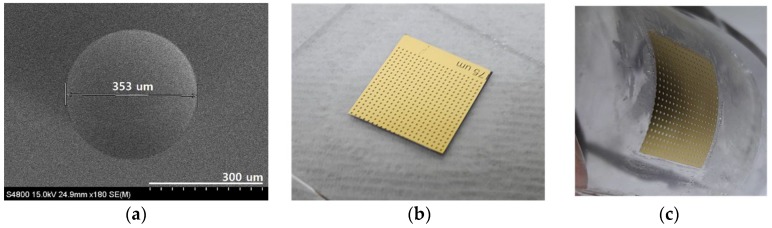
The fabricated microlens array (**a**) implemented using thermal reflow process, (**b**) firstly molded with PDMS on glass substrate and (**c**) secondly molded with UV curing resin on PET film.

A cylindrical photoresist structure with a height of 10 μm and a diameter of 350 μm was thermal-reflowed. The microlens mold with a curvature of 778.9 μm (r_sph_ was 175 μm and h_sph_ was 19.91 μm) was fabricated, as shown in Figure 4a. The focal length of the microlens implemented using this thermal-reflowed mold was 1.442 mm. Spacing among every microlens was fabricated as 500 μm.

Figure 4b shows the concave microlens array and the gold layer that was molded in PDMS on a cover slip glass substrate. By using the UV-imprinting process, the concave pattern was molded in NOA 72 on PET film (Figure 4c).

**Figure 5:**
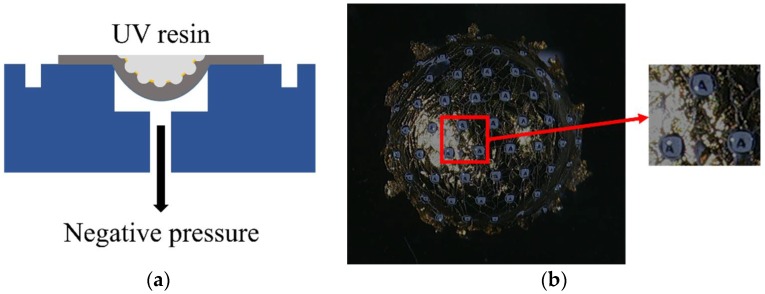
(**a**) The fabrication schematic for the hemispherical substrate. (**b**) The fabricated microlens array with the screening layer on the hemispherical substrate.

One of the advantages of our PLISL process is that three-dimensional microstructure and integrated layer is transferred not only on a planar substrate but also on a hemispherical substrate using the widely known negative pressure technique (Figure 5a). Due to the negative pressure, the flexible PDMS mold is deformed as the hemispherical structure. Figure 5b shows a microlens array on the hemispherical substrate using a three-dimensionally printed chamber.

### 3.2. Integrated Imaging System with a Conventional CCD Image Sensor

**Figure 6:**
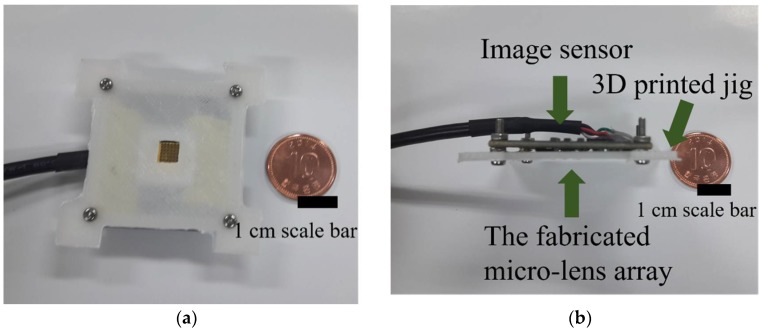
(**a**) The fabrication schematic for the hemispherical substrate. (**b**) The fabricated microlens array with the screening layer on the hemispherical substrate.

Figure 6 shows the assembled imaging system with the microlens array adjusted by the 3D printed jig, springs, and bolts. Due to the springs and the bolts, the distance between the microlens array and the CCD sensor was calibrated to focus every microlens’ image. The light-entering window of the 3D printed jig is a square shape of 4 × 4 mm. Eighteen microlenses receive light from the light-entering window. The photo sensing part of the CCD sensor is a rectangular shape that is 5.37 × 4.04 mm and 1920 × 1080 pixels.

**Figure 7:**
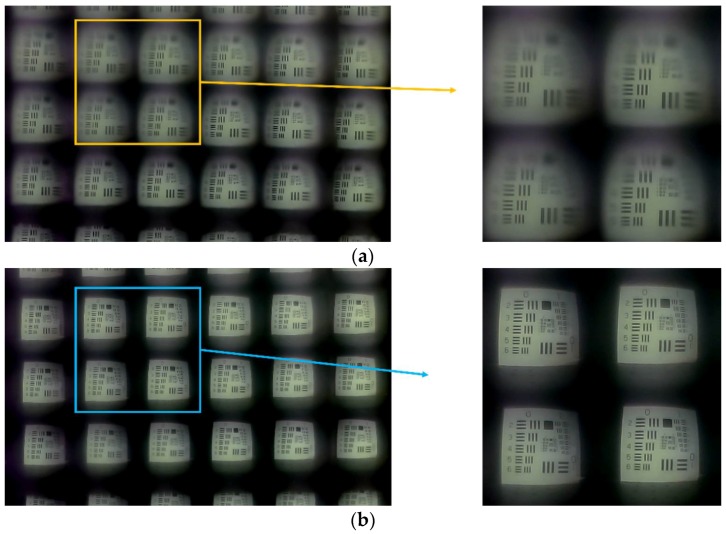
The captured images of the printed USAF 1951 test pattern at a distance of 10 cm by our imaging system with (**a**) the microlens-facing-outside setup and (**b**) the microlens-facing-inside setup.

Figure 7 presents images of a printed USAF 1951 pattern captured by two different setups: the microlens-facing-outside setup (Figure 7a) and the microlens-facing-inside setup (Figure 7b). The facing-inside setup shows better contrast around the rim than the looking-outside setup. We could assume that the apertures of the facing-inside setup play a better role because the gold screening layer of the facing-inside setup is closer to the image sensor by a thickness of the NOA substrate.

**Figure 8:**
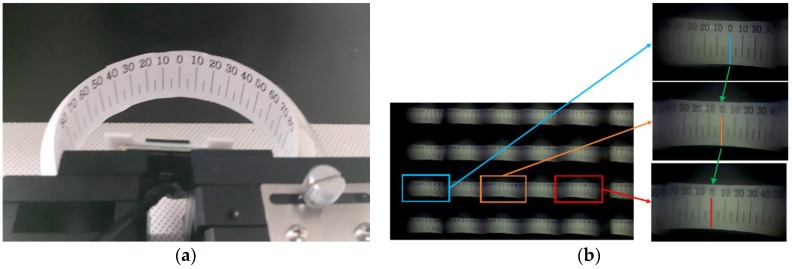
The experimental setup for measuring the FOV of the assembled imaging system. (**a**) The captured image represents about 100 degrees of the FOV. (**b**) The captured images of (upper) the leftmost microlens, (middle) the middle microlens, and (lower) the rightmost microlens.

Field-of-view (FOV) of the assembled imaging system was measured using a 3D-printed half-circle structure and a paper-printed goniometer, as shown in Figure 8a. Figure 8 shows that the leftmost microlens captured −50 degrees and the rightmost microlens took +50 degrees. This means that our imaging system has about 100 degrees of FOV, which is higher than the FOV of the single microlens (80 degrees).

**Figure 9:**
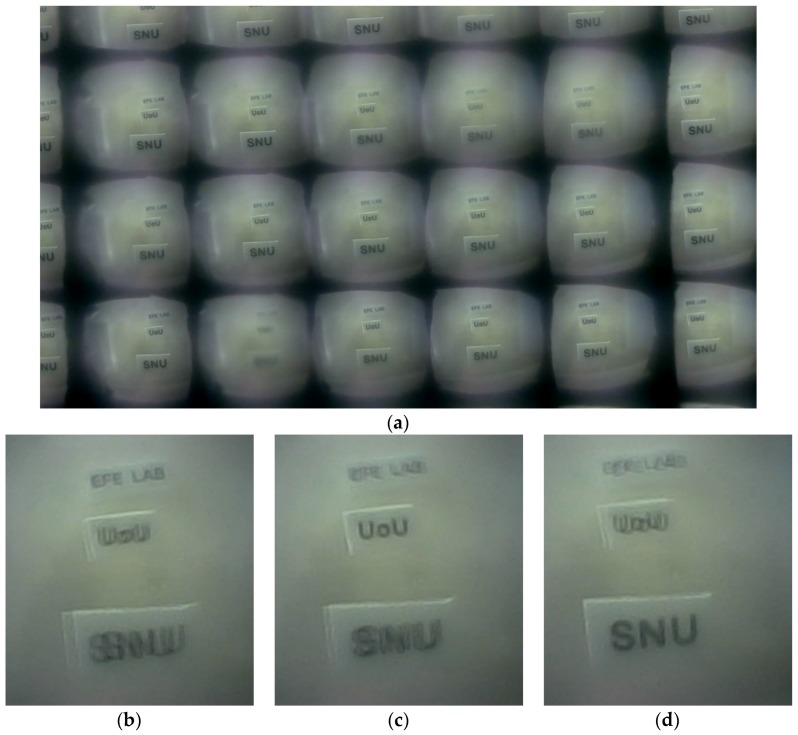
(**a**) The captured 18 subimages. (**b**) The reconstructed image focusing the “EFE LAB” apart 3 cm from the imaging system. (**c**) The reconstructed image focusing the “UoU” apart 2 cm from the imaging system. (**d**) The reconstructed image focusing the “SNU” apart 1 cm from the imaging system.

Every subimage represents different perspective views of the identical object. Due to this plentiful information, post-image processing of the multiaperture imaging system generates diverse images including higher-resolution images [21] and multifocusing images [22]. Figure 9 shows multifocusing images. Using the 18 subimages of one single shot (Figure 9a), three differently focused images were reconstructed, as shown in Figure 9b–d. The characters “SNU”, “UoU”, and “EFE LAB” are apart 1 cm, 2 cm, and 3 cm from the imaging system, respectively.

## 4. Discussion & Conclusions

The imaging system mimicking an insect’s compound eye was developed. Due to the light screening layer among the microlenses fabricated by our PLISL process and the adjustable jig, all of the 18 subimages were clearly focused. A simple change in the mask for the microlens patterning can increase the number of the subimages, which means that there is higher angular resolution and depth resolution in reconstructed images. Although the field-of-view of our imaging system is wider than other types of microlens array imaging systems and a conventional 35 mm camera with a compact lens system, it should be increased for recent emerging demands such as virtual reality and unmanned vehicles. Currently, we are researching a microlens array on hemispherical light sensors to meet these requirements.

## Supplementary Materials

The following are available online at http://www.mdpi.com/1424-8220/18/7/2011/s1.
