# Supplementary material for: Insect-Mimetic Imaging System Based on a Microlens Array Fabricated by a Patterned-Layer Integrating Soft Lithography Process
Source: Sensors (Basel). 2018 Jun 22;18(7):2011. doi: 10.3390/s18072011 (PMC6068472; doi:10.3390/s18072011)
Supplement: Supplementary file 1 [file sensors-18-02011-s001.pdf]

sensors

Supplementary Information

Insect-Mimetic Imaging System Based on a Microlens Array Fabricated by a Patterned-Layer Integrating Soft Lithography Process

Minwon Seo [1], Jong-Mo Seo [1,2,*], Dong-il "Dan" Cho [1] and Kyoin Koo [3,*]

[1] Department of Electrical and Computer Engineering, Seoul National University, Seoul 08826, Korea; sm7bmw@snu.ac.kr (M.S.); dicho@snu.ac.kr (D.C.)
[2] Biomedical Research Institute, Seoul National University Hospital, Seoul 03080, Korea
[3] Department of Biomedical Engineering, University of Ulsan, Ulsan 44610, Korea
* Correspondences: callme@snu.ac.kr (J.-M.S.); kikoo@ulsan.ac.kr (K.K.); Tel.: +82-2-880-1739 (J.-M.S.)

Received: 16 May 2018; Accepted: 21 June 2018; Published: date

Numerical aperture of fabricated micro-lens

We calculated for several sizes and concluded that cylindrical structure (before thermal reflow) with the radius of 175 um and the height of 10 um had suitable numerical aperture to focus the image to the CCD image sensor with a pixel size of 3 um. Due to the protecting glass, the focal length of the micro-lens should be above 670 um. Considering the thickness of the micro-lens layer (about 200 um) and focusing distance afterwards, the focal length should be generously determined. We determined 1.2 mm as the focal length of the micro lens. There are plenty of models to get the focal length of 1.2 mm. Three different models were calculated based on the height (H) of the cylindrical structure. As the H increases, the corresponding numerical aperture (NA) increases. For H = 5 um, NA = 0.095. For H = 10 um, NA = 0.134. For H = 20 um, NA = 0.186. In this paper, the NA of the micro-lens was 0.125, because the pixel size of the CCD image sensor was 3 um x 3 um (AR0230CS, ON Semiconductor, USA). The height (h) and the focal length (f) of micro-lens are calculated using equation (3), (4) in a manuscript file.

Table shows the minimum spot size and numerical aperture of the micro-lens when the radius of the micro-lens is fixed to 175 um. Figures show the changes in the numerical aperture and the focal length as the radius of the micro-lens increases. There is inversion relation between the numerical aperture and the focal length.

Table S1. Calculation of minimum spot size as height of cylinder increases when the radius of the micro-lens is 175 um.

H (um)	NA	Beam waist (um)	Diameter of minimum spot size (um)
5	0.095	1.877	3.755
10	0.134	1.331	2.662
20	0.186	0.959	1.918

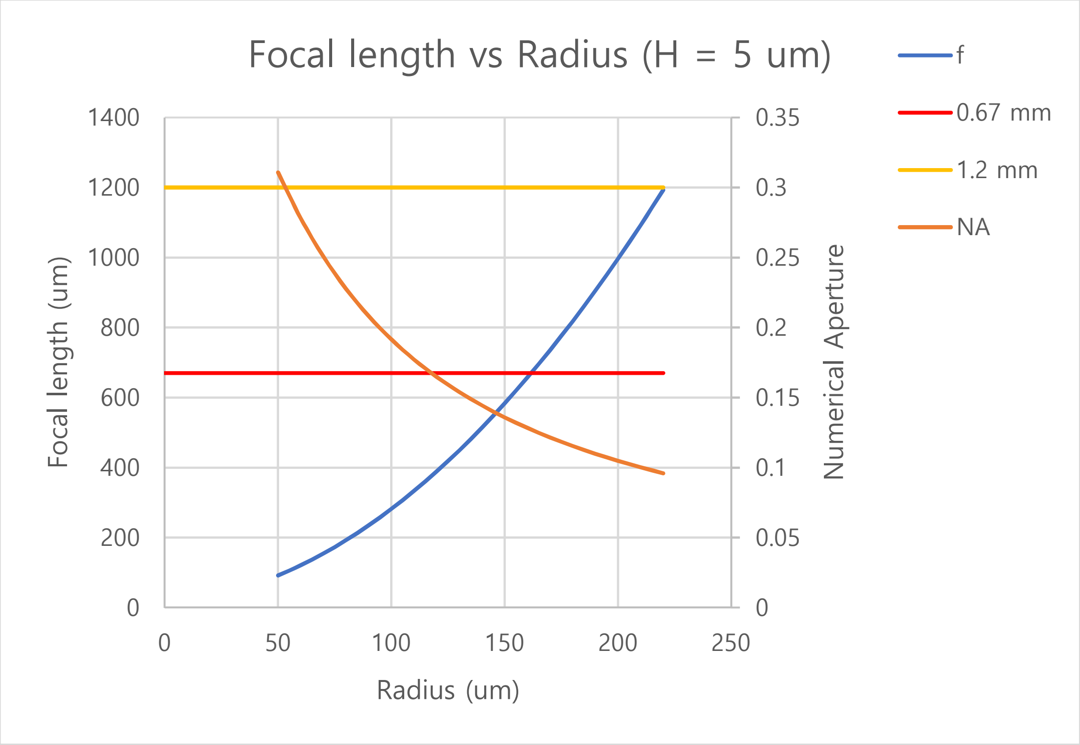

Figure S1. Changes in focal length and numerical aperture as radius increases when H = 5 um. Red and yellow lines are reference line. Red line refers to minimum focal length and yellow line, desired focal length in this paper.

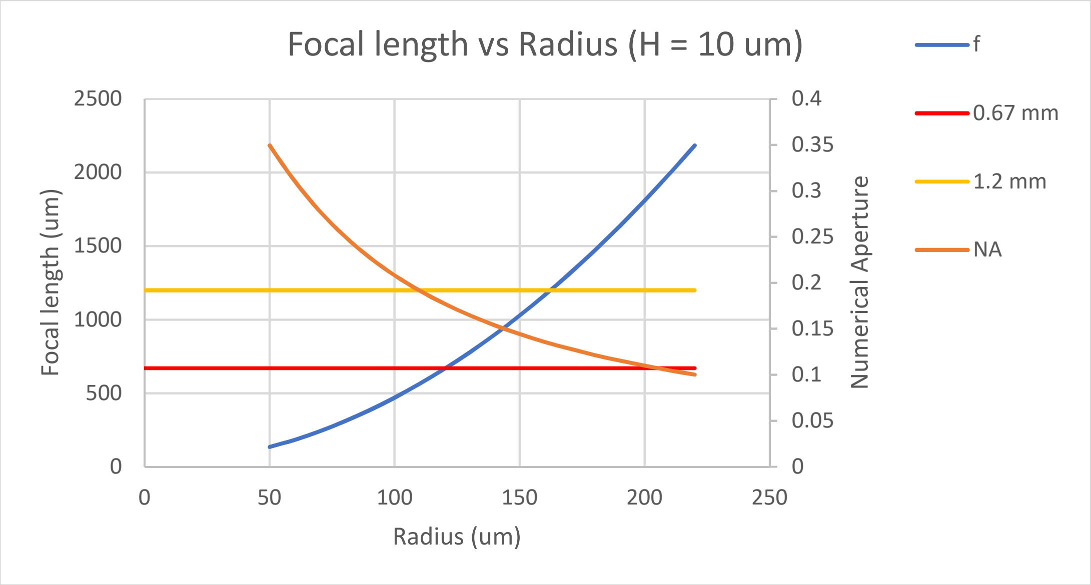

Figure S2. Changes in focal length and numerical aperture as radius increases when H = 10 um. Red and yellow lines are reference line. Red line refers to minimum focal length and yellow line, desired focal length in this paper.

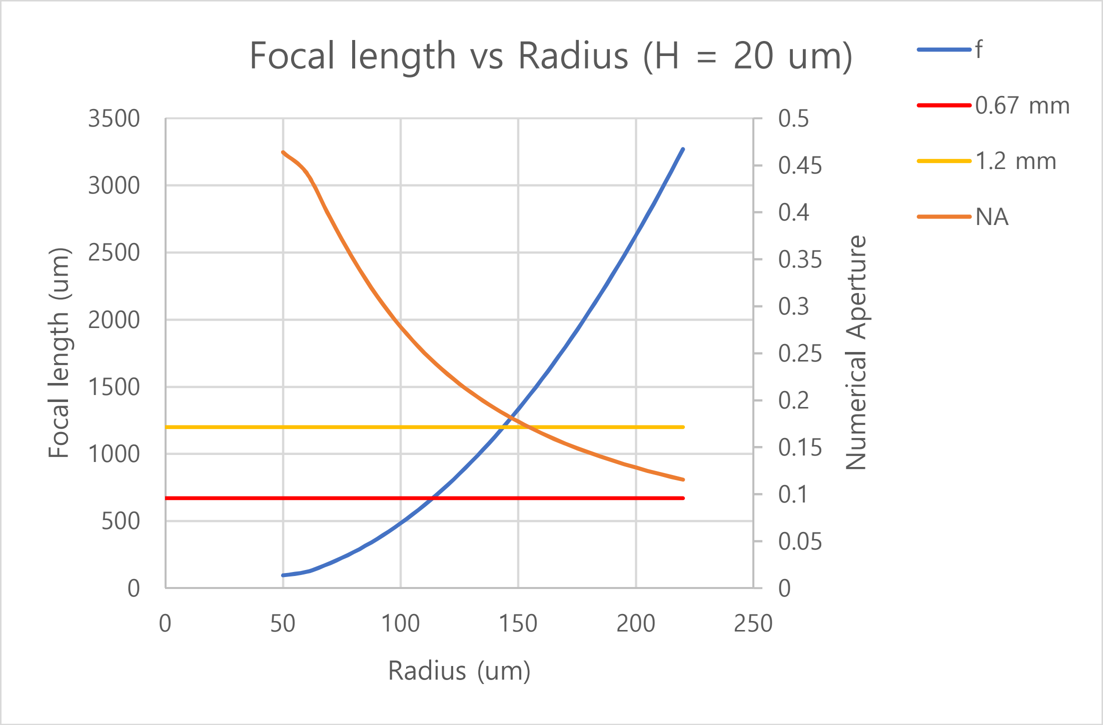

Figure S3. Changes in focal length and numerical aperture as radius increases when H = 20 um. Red and yellow lines are reference line. Red line refers to minimum focal length and yellow line, desired focal length in this paper.
